# Exciton-driven Floquet engineering from first principles

**DOI:** 10.1038/s41535-026-00951-y

**Published:** 2026-10-28

**Authors:** Ruipeng Li, Benshu Fan, Umberto De Giovannini, Hannes Hübener, Angel Rubio

**Affiliations:** 1 Max Planck Institute for the Structure and Dynamics of Matter, Center for Free Electron Laser Science, Hamburg, Germany; 2 Università degli Studi di Palermo, Dipartimento di Fisica e Chimica Emilio Segrè, Palermo, Italy; 3 Initiative for Computational Catalysis, The Flatiron Institute, Simons Foundation, New York City, NY USA

**Keywords:** Materials science, Physics

## Abstract

Floquet engineering uses periodic driving to tailor electronic structures and material properties out of equilibrium. Excitons offer a distinct route to this control: their electron-hole dynamics can act as an internal drive, enabling Floquet engineering beyond direct laser dressing. A microscopic description of this regime requires simultaneous access to excitonic dynamics and their momentum-resolved photoemission signatures, which remains challenging in realistic materials. Here we develop an efficient first-principles framework combining real-time time-dependent density functional theory with a modified long-range-corrected exchange-correlation kernel, Floquet-band analysis, and time- and angle-resolved photoemission simulations. Using monolayer hexagonal boron nitride as a benchmark, we capture a subgap excitonic resonance and show that the resulting oscillatory excitonic field generates clear Floquet sidebands and band renormalization, even without temporal overlap between pump and probe pulses. Our approach establishes a practical and computationally efficient route for predicting nonequilibrium Floquet phenomena driven by collective excitations in quantum materials.

## Introduction

Controlling the electronic structure of quantum materials on ultrafast time scales is a central goal of nonequilibrium materials science^1–3^. Floquet engineering offers a particularly powerful route to this goal by using coherent periodic motion to create effective electronic structures that are not available in equilibrium. Traditionally, this approach has employed time-periodic driving fields to dress Bloch electrons^4,5^, which can dynamically reshape electronic structures^6–20^ and optical responses^21–26^, while also generating novel quantum states^27–31^. In particular, time- and angle-resolved photoemission spectroscopy (TrARPES) has emerged as a powerful tool for directly resolving transient Floquet band structures and dynamical processes in several representative quantum materials, including (antiferromagnetic) topological insulators^32–36^, graphene^37–39^, black phosphorus^40–44^, and layered chalcogenides^45–48^. More broadly, Floquet engineering can be viewed as involving two key ingredients: the source of the periodic drive and the degrees of freedom dressed by it. From this perspective, the driving source need not be an external laser field, but may also arise internally from collective bosonic excitations in solids, such as coherent phonons^49^ and excitons^50^. Experimentally, exciton-driven Floquet engineering has recently been reported in monolayer *WS*_2_^51^. A recent model study based on the dynamical projective operatorial approach likewise showed that resonant pumping can sustain coherent oscillations of the excitonic order parameter after the pump pulse, generating sidebands distinct from those produced directly by the external field^52^. In addition, the dressed object can extend beyond Bloch states to exciton states^53,54^, while excitons can further interact with and reshape Floquet states^55–57^. Taken together, these developments show that excitons, as correlated electron-hole excitations, provide a particularly intriguing route for exploring Floquet physics beyond the conventional single-particle picture. In this work, we focus on the regime in which excitons act as an intrinsic time-periodic driving source that induces Floquet physics.

Describing exciton-driven Floquet spectra remains a substantial theoretical challenge because it involves two intertwined tasks. The first is the description of excitons themselves^58^. In first-principles calculations, this is commonly achieved either within quasiparticle-based many-body perturbation theory, such as the GW plus Bethe Salpeter equation (GW-BSE)^59,60^, or within time-dependent density functional theory (TDDFT) using modified exchange-correlation (xc) kernels with appropriate long-range contributions to capture excitonic effects^61–68^. The second is the simulation of ultrafast photoemission spectra. This can be formulated through nonequilibrium Green-function approaches, as in ab initio time-dependent adiabatic GW (TD-aGW)^50,69^, or through real-time wavefunction-based methods combined with explicit photoelectron extraction schemes, such as the time-dependent surface flux method in the semi-periodic system (t-SURFFP)^70,71^. While these approaches can describe excitonic properties at different levels, they differ in computational cost and practical applicability. It is therefore highly desirable to develop a balanced strategy that retains the essential first-principles character of the problem while remaining efficient enough for realistic simulations of exciton-driven Floquet band structures and their TrARPES signatures.

**Fig. 1:**
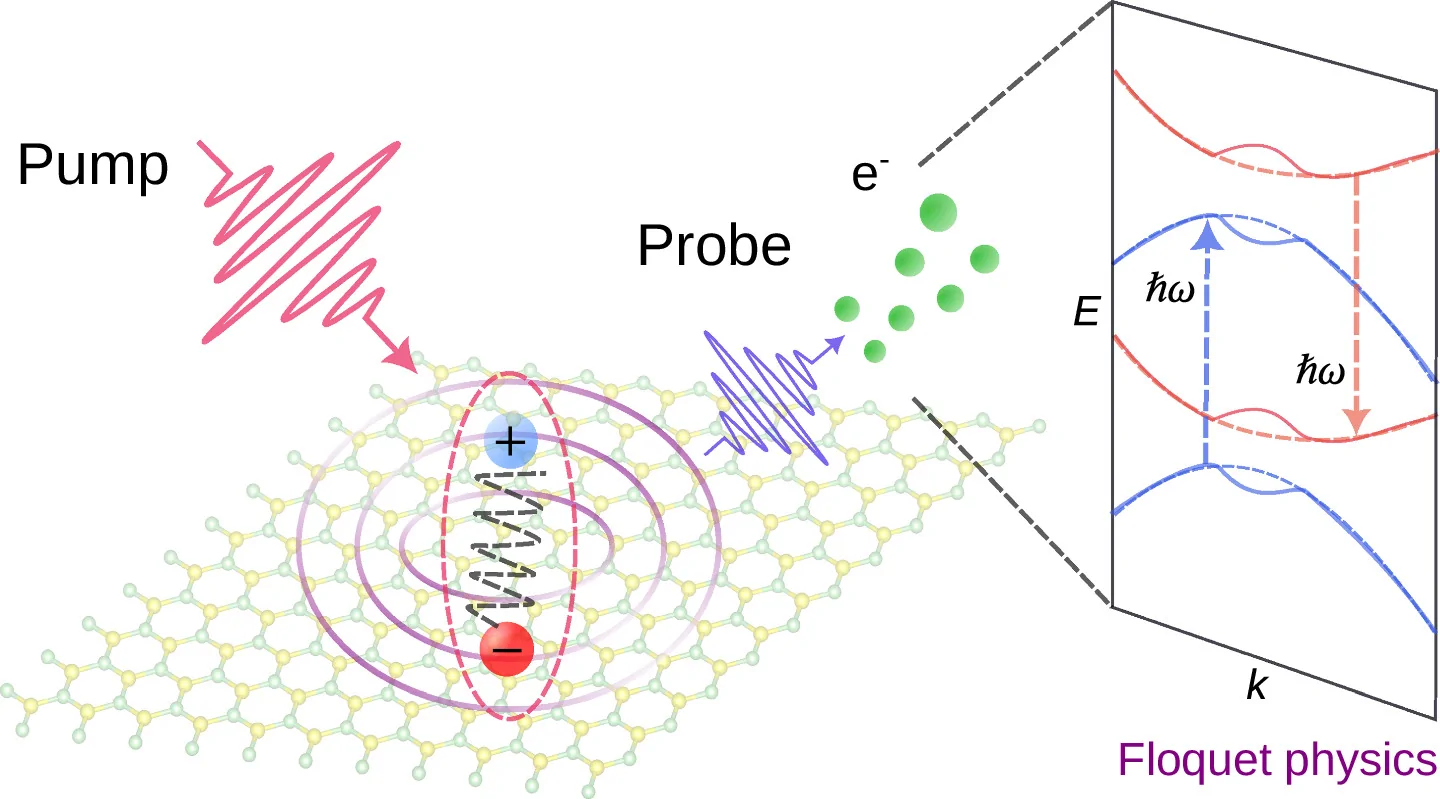
Schematic of exciton-driven Floquet physics. A pump pulse induces an excitonic excitation in the material. The resulting time-dependent excitonic response acts as an internal periodic drive that dresses the underlying electronic bands. A time-delayed probe pulse photoemits electrons from the driven material, enabling measurement of the nonequilibrium Floquet spectrum. The periodic excitonic drive generates Floquet replicas displaced by integer multiples of the characteristic excitonic driving energy, *ℏω*.

In this manuscript, we apply the modified long-range-corrected (MLRC) xc kernel^67,68^ implemented in our open-source OCTOPUS code^72^ to describe ultrafast exciton dynamics within real-time TDDFT. Taking monolayer hexagonal boron nitride (hBN) as a benchmark system, we first calculate the optical absorption spectra and identify a pronounced subgap excitonic resonance, and then simulate the excitonic-field-induced TrARPES intensity plots and Floquet band structures, as shown in Fig. 1. For hBN, we identify clear exciton-induced sidebands in the photoemission spectra, together with Floquet band renormalization, even without temporal overlap between the pump and probe pulses. Our approach provides a balanced framework that combines accuracy and efficiency for describing exciton-driven Floquet phenomena and their momentum-resolved photoemission fingerprints, without resorting to a full nonequilibrium Green-function treatment. More broadly, it offers a promising foundation for future extensions to more complex nonequilibrium situations and coupled degrees of freedom.

## Results

### Excitonic resonance in monolayer hBN

Before investigating exciton-induced Floquet features in the pump-probe simulations, it is necessary to first characterize the equilibrium optical response of monolayer hBN and determine the relevant excitonic energy scale. The optical absorption spectrum provides a direct measure of the neutral electronic excitations of the system and therefore serves as an essential benchmark for evaluating whether the employed exchange-correlation treatment adequately captures long-range electron-hole interactions.

**Fig. 2:**
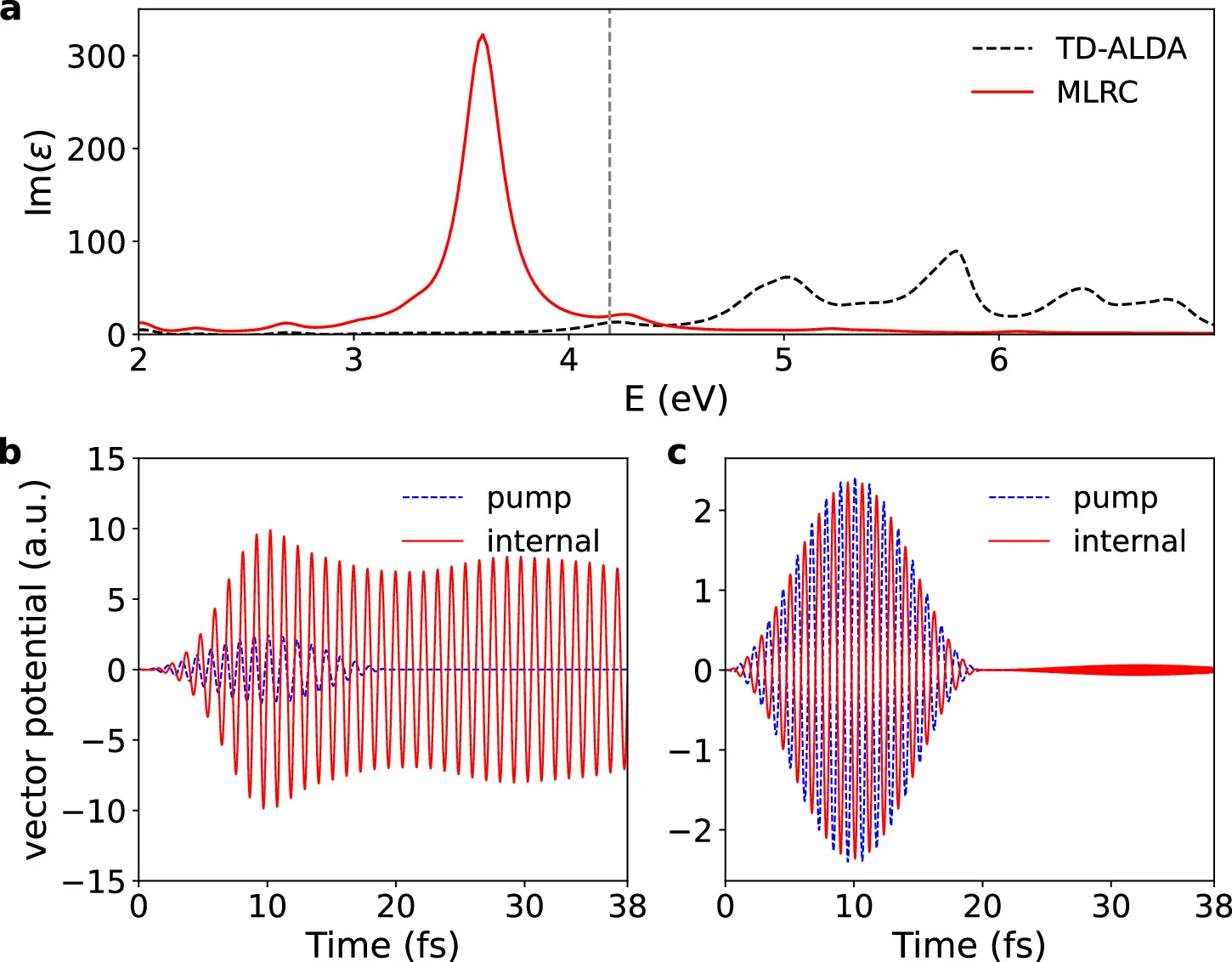
Optical response of hBN. **a** Optical absorption spectra calculated within real-time TDDFT using the MLRC and TD-ALDA approaches. The MLRC calculation exhibits a pronounced excitonic resonance at approximately 3.6 eV, below the Kohn-Sham band gap, whereas the TD-ALDA spectrum contains only continuum onsets above the absorption edge. The vertical gray dashed line marks the calculated band-gap energy. **b** Time evolution of the external pump vector potential, **A**_pu_(*t*), and the corresponding internal xc vector potential, **A**_xc_(*t*), in the resonantly driven MLRC calculation. After the external pump vanishes, **A**_xc_(*t*) continues to oscillate with a sizable amplitude generated by the resonant excitation. **c** Corresponding time evolution obtained within TD-ALDA under otherwise identical pumping conditions. In the absence of the long-range electron-hole interaction, the induced macroscopic vector potential **A**_ind_(*t*) rapidly decays after the pump pulse, and no persistent oscillating internal field is sustained. The weak increase after *t* = 20fs originates from the probe-induced current response.

In Fig. 2a, we show the optical spectra of monolayer hBN calculated using real-time TDDFT within the MLRC and time-dependent adiabatic local density approximation (TD-ALDA)^73^ methods. The calculations follow the scheme described in Methods. The TD-ALDA spectrum exhibits a weak absorption onset near the marked band-gap energy at approximately 4.19 eV, followed by several broad interband-transition features between 5 and 7 eV. In contrast, the MLRC calculation produces an intense and narrow peak at about 3.6 eV, well below the TD-ALDA absorption edge. The much weaker structures below the dominant excitonic resonance, including those near  ~ 2.0 and  ~ 2.7 eV, arise from finite-time Fourier-transform effects and are not assigned to additional physical excitonic resonances. This pronounced redistribution of oscillator strength from the higher-energy interband continuum to a subgap resonance is characteristic of a strongly bound exciton arising from the effective long-range electron-hole interaction included through the MLRC exchange-correlation field. Relative to the marked gap, the position of this resonance suggests an exciton binding energy of roughly 0.59 eV. The substantial enhancement of the low-energy peak and the simultaneous suppression of the higher-energy structures demonstrate that electron-hole interactions qualitatively modify the optical response of hBN and cannot be captured within the TD-ALDA description. Our results are qualitatively consistent with previous GW-BSE calculations^74^, supporting the validity of the calculated excitonic spectral features.

### Photoemission fingerprints of exciton-driven Floquet states

We then perform TrARPES simulations for monolayer hBN as a representative demonstration, considering both resonant and off-resonant excitation conditions. For the resonant case, calculations are performed using both the MLRC and TD-ALDA approaches to elucidate the role of excitonic effects in the TrARPES spectral features. To generate the excitonic polarization, the system is driven by an *x*-polarized pump pulse with a photon energy of *ℏω*_pu_ = 3.6eV, resonant with the exciton energy, and a duration of *T*_pu_ = 20fs. After the pump pulse has ended, the system is probed by a weak field with a photon energy of *ℏω*_pr_ = 40.8eV and a duration of *T*_pr_ = 24fs. This probe energy follows the established t-SURFFP benchmark for monolayer hBN^71^ and is also readily accessible in TrARPES experiments^75^. For comparison, the TD-ALDA reference calculation is performed under identical pump-probe conditions. As shown in Fig. 2b and c, the pump-induced MLRC exchange-correlation vector potential, **A**_xc_, continues to oscillate after the pump pulse has ended. By contrast, in the TD-ALDA calculation, **A**_ind_ decays rapidly, leaving only a weak response induced by the probe pulse. These results demonstrate that resonant excitation generates a pronounced excitonic polarization, which in turn produces persistent oscillations of **A**_xc_. In the following, we show how this exciton-induced exchange-correlation field can drive Floquet physics in the material.

**Fig. 3:**
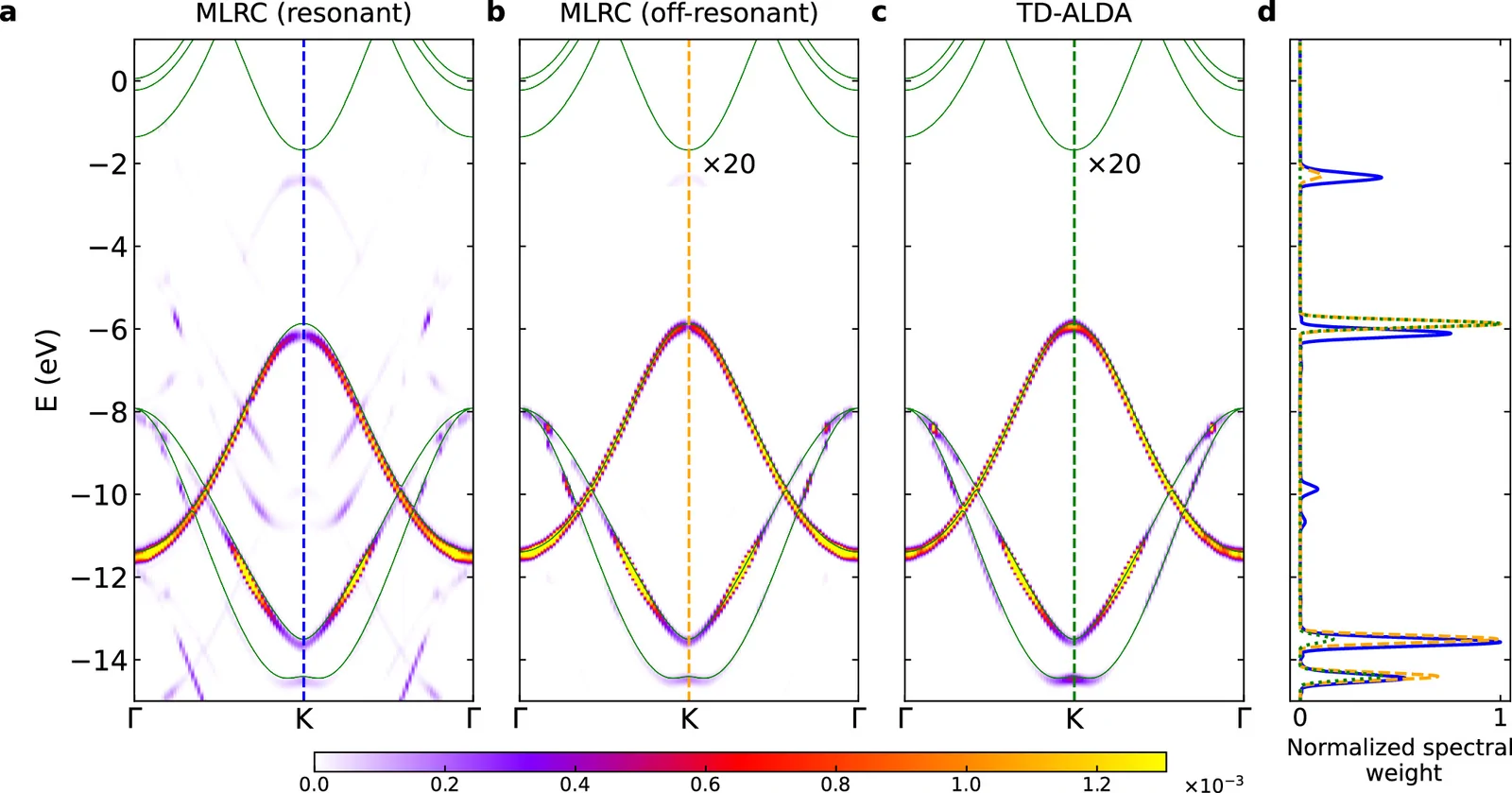
Photoemission spectra of exciton Floquet states in monolayer hBN. **a**–**c** Pump-probe TrARPES spectra along the Γ − K − Γ momentum path calculated for a, resonant excitation within the MLRC approach; b, off-resonant excitation within MLRC; and c, resonant excitation within TD-ALDA. The color scale represents the photoemission spectral weight, while the green solid curves indicate the corresponding equilibrium band structure. For clarity, the spectral intensities in (**b**) and (**c**) are multiplied by a factor of 20 for energies above  − 2.5e*V*. Under resonant excitation, the MLRC spectrum exhibits pronounced additional spectral weight inside the equilibrium band gap, forming a dispersive replica of the valence band together with substantial band renormalization around the K point. These features are strongly suppressed under off-resonant excitation and are essentially absent in the TD-ALDA calculation. d Normalized TrARPES spectral weight evaluated at the K point as a function of energy for the resonant MLRC (blue solid line), off-resonant MLRC (orange dashed line), and TD-ALDA calculations (green dotted line). These curves correspond to the spectral weight along the vertical dashed lines at the K point in panels a, b and c, respectively. The energy range is chosen to be the same as that used in the TrARPES spectra for comparison.

As shown in Fig. 3a and b, the TrARPES spectra along Γ − K − Γ calculated using the MLRC approach exhibit an additional replica of the valence band, separated from the original band by approximately the exciton energy. Because the pump and probe pulses do not overlap in time, this feature can be identified as a Floquet sideband generated by the persistent excitonic field rather than by the external pump field itself. In contrast, the TD-ALDA spectrum in Fig. 3c displays appreciable spectral weight only near the equilibrium band structure and shows no clear feature within the band gap. This reflects the inability of the TD-ALDA to describe exciton-driven Floquet physics because it lacks the long-range electron-hole interaction required to sustain excitonic polarization. Notably, the MLRC spectrum also exhibits substantial band renormalization near the valence-band maximum (VBM) at the K point and its Floquet replica, indicating strong hybridization between the conduction- and valence-band replicas. For the off-resonant case, with *ℏω*_pu_ = 3.2e*V*, the MLRC TrARPES spectrum shown in Fig. 3b contains a considerably weaker VBM Floquet replica than that observed under resonant excitation, indicating that the off-resonant pump generates a much weaker oscillating excitonic polarization. The resulting exciton-induced Floquet response is therefore strongly suppressed, and the associated sideband and band-renormalization features become much less pronounced.

To quantify these observations, in Fig. 3d we show the spectral weight of TrARPES at the K point for resonant MLRC, off-resonant MLRC and TD-ALDA. Because the spectra are normalized, the comparison primarily reflects peak positions and the relative redistribution of spectral weight within each calculation, rather than differences in the absolute photoemission intensity. The TD-ALDA spectrum is dominated by a single narrow peak near  − 5.9e*V*, corresponding to the equilibrium valence-band state, and exhibits essentially no spectral weight within the band-gap region. The off-resonant MLRC result retains a similar principal valence-band peak and develops only a weak satellite near  − 2.3e*V*, indicating that the off-resonantly generated excitonic polarization produces only a small Floquet response. In contrast, resonant excitation leads to a pronounced redistribution of spectral weight: the dominant valence-band peak shifts toward a larger binding energy, to approximately  − 6.2e*V*, while a strong additional peak emerges near  − 2.6e*V*. The energy separation is comparable to the characteristic excitonic driving energy, supporting the interpretation of the higher-energy feature as an exciton-field-induced Floquet replica. The simultaneous displacement and partial splitting of the valence-band feature further indicate strong hybridization and level repulsion between the original band and its exciton-induced replicas. Overall, the comparison demonstrates that resonant excitation greatly enhances the excitonic polarization and produces substantially stronger Floquet sidebands and band renormalization than either off-resonant excitation or the TD-ALDA treatment.

### Floquet-band analysis of excitonic dressing

**Fig. 4:**
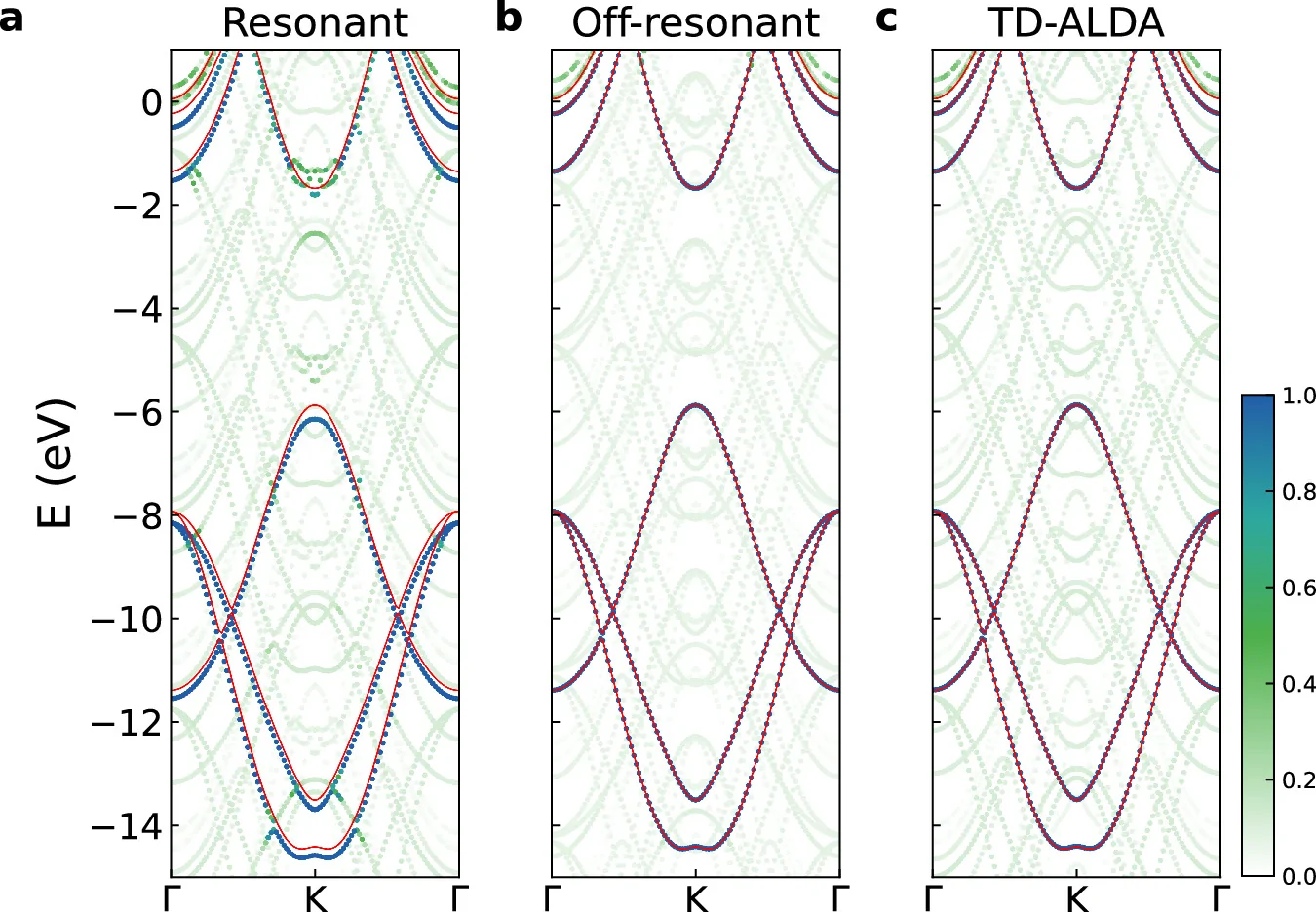
Exciton-dressed Floquet band structure of monolayer hBN. **a**–**c** Floquet band structures along the Γ − K − Γ momentum path extracted from the pump-probe simulations for **a**, resonant excitation within the MLRC approach; **b**, off-resonant excitation within MLRC; and **c**, resonant excitation within TD-ALDA. The red solid curves denote the equilibrium DFT band structure, while the colored points represent the Floquet quasienergy states; the color map indicates the corresponding Floquet spectral weight. Under resonant excitation, pronounced Floquet replicas appear throughout the spectrum, including a clearly visible valence-band replica inside the equilibrium band gap. Significant deviations of the strongly weighted Floquet branches from the equilibrium bands, particularly around the K point, indicate substantial band renormalization driven by the persistent excitonic polarization. In the off-resonant case, the in-gap replica remains visible but carries considerably less Floquet weight, while the underlying bands remain close to their equilibrium dispersions, indicating much weaker Floquet dressing and negligible band renormalization. In contrast, within the TD-ALDA, the spectral weight is concentrated almost entirely on the zeroth-order Floquet bands, with higher-order replicas strongly suppressed because no excitonic field persists after the pump pulse.

To further relate our TrARPES simulation results to the underlying Floquet quasienergy structure, we compare them with the Floquet band structure calculated directly using the real-time Floquet-analysis framework implemented in OCTOPUS^20^. Although the Floquet analysis and TrARPES simulation are performed within the same real-time propagation, they provide complementary information: the former directly resolves the intrinsic quasienergy branches and their Floquet weights, whereas the latter includes the probe-dependent photoemission process and therefore determines how these dressed states appear in an experimentally accessible spectrum. In Fig. 4, the Floquet band structure extracted from the resonant pump-probe simulation exhibits a clear in-gap sideband that is absent from the equilibrium density functional theory (DFT) band structure. The red curves represent the equilibrium Kohn-Sham bands, while the colored points denote the Floquet quasienergy states generated by the time-periodic excitonic field. Around the K point, the zeroth-order Floquet replica of the VBM lies near  − 6.2e*V*, whereas an additional Floquet branch appears inside the band gap at approximately  − 2.6e*V*. This branch closely reproduces the momentum dispersion of the valence band and is shifted upward by about 3.6 eV, in agreement with the exciton energy. It can therefore be identified as the first-order exciton-induced Floquet replica of the valence band. The relatively strong Floquet weight of this branch near K, together with its proximity to the equilibrium conduction band, suggests appreciable coupling between the exciton-shifted valence-band replica and the conduction states, which may account for the spectral-weight redistribution and band renormalization observed in the corresponding TrARPES spectrum. Because the Floquet analysis is performed during the probe interval after the external pump has ended, the in-gap replica provides direct evidence that the persistent excitonic polarization acts as an internal periodic driving field and sustains Floquet dressing of the electronic structure. Weaker light green branches at other energies can be attributed to additional Floquet replicas with smaller spectral weights.

For comparison, the Floquet band structures along the Γ − K − Γ momentum path obtained under off-resonant excitation and within the TD-ALDA exhibit substantially weaker dressing effects, as shown in Fig. 4. In the off-resonant MLRC calculation, a faint replica of the valence band can still be identified inside the equilibrium band gap, but its Floquet weight is markedly reduced relative to the resonant case. Moreover, the equilibrium bands and their replicas remain nearly unchanged, indicating only weak hybridization and negligible band renormalization. This behavior is consistent with the weaker residual excitonic polarization generated by the off-resonant pump. In the TD-ALDA calculation, by contrast, essentially only the zeroth-order Floquet bands survive, while the higher-order replicas are strongly suppressed. Because the TD-ALDA lacks the long-range electron-hole interaction required to form and sustain an excitonic polarization after the pump pulse, no persistent internal periodic field remains to dress the electronic states. The comparison therefore confirms that the in-gap sidebands and band renormalization observed under resonant excitation originate predominantly from exciton-induced Floquet dynamics rather than from the external pump field itself.

## Discussion

In this work, we investigate exciton-driven Floquet physics in monolayer hBN from first principles, following the evolution from coherent excitonic polarization to Floquet dressing and its momentum-resolved photoemission signatures. Treating exciton dynamics and Floquet response within the same real-time framework establishes a direct connection between the internal excitonic field, Floquet sidebands, and band renormalization.

By comparing the resonant and off-resonant MLRC calculations with the TD-ALDA reference calculation, we identify the microscopic origin of the observed Floquet features. Under resonant excitation within MLRC, the exchange-correlation vector potential continues to oscillate after the external pump has vanished, providing an internal periodic drive that produces pronounced in-gap Floquet sidebands, redistribution of TrARPES spectral weight, and band renormalization through hybridization with exciton-induced replicas. In contrast, off-resonant pumping generates only a weak residual excitonic polarization, yielding faint in-gap replicas and little band renormalization, while in the TD-ALDA reference calculation the higher-order Floquet replicas are strongly suppressed because the long-range electron-hole interaction required to sustain the excitonic polarization is absent. The agreement between the TrARPES signatures and Floquet bands extracted from the same propagation provides a consistent microscopic picture of this excitonic-field-driven dressing, while the explicit photoemission simulation additionally establishes how the underlying Floquet states manifest in a probe-dependent experimental observable. Taken together, these results show that, during the probe interval, the relevant periodic drive is the persistent internal excitonic field rather than the external pump.

Methodologically, our framework provides an efficient first-principles route for accessing exciton-driven Floquet phenomena and their momentum-resolved signatures. By combining real-time TDDFT with the MLRC exchange-correlation field, t-SURFFP photoemission simulations, and direct Floquet analysis within OCTOPUS, excitonic dynamics, TrARPES spectra, and Floquet states can be obtained consistently from the same time propagation without a full nonequilibrium Green-function treatment. This balance of computational efficiency, physical transparency, and direct access to experimentally relevant observables makes the approach suitable for extending exciton-driven Floquet studies to more complex materials.

Despite its computational efficiency, the present MLRC approach has important limitations in its description of the excitonic spectrum. In particular, because the long-range electron-hole interaction is represented through a macroscopic exchange-correlation vector potential characterized by only a small number of material-dependent parameters, the method is primarily capable of reproducing a single dominant bound-exciton resonance rather than the full manifold of excitonic states. Consequently, systems containing several bound excitons cannot in general be described with the same level of microscopic resolution. Moreover, as generally found for long-range-corrected kernels^76^, obtaining a physically reasonable exciton binding energy can be accompanied by an overestimation of the excitonic oscillator strength. Accordingly, the height of the excitonic peak in Fig. 2a should not be regarded as quantitatively accurate. Since the strength of the exciton-driven Floquet response depends on the pump-induced excitonic polarization, this limitation may also lead to an overestimation of the spectral weight of the Floquet sidebands and the magnitude of the associated band renormalization. These quantities should therefore be interpreted primarily at the qualitative level rather than as quantitative predictions of their absolute magnitudes. A further limitation arises for materials with strongly anisotropic excitonic responses, where the exciton binding energy and oscillator strength depend sensitively on crystallographic direction. In such cases, a simple macroscopic MLRC parametrization may not be sufficiently flexible to capture the full directional dependence of the electron-hole interaction and the resulting anisotropic exciton spectrum.

Building upon the present MLRC framework, several extensions could further enhance its predictive capability. In particular, combining hybrid or screened-exchange functionals^77,78^ with the t-SURFFP and real-time Floquet-analysis approaches could provide a more accurate description of the exciton dynamics and the corresponding Floquet physics in real materials. Applying such an extended framework to systems hosting anisotropic excitons^79^ and valley-selective excitations^80^ could uncover additional pathways for controlling nonequilibrium electronic structure through internally generated collective fields. Moreover, the present real-time TDDFT simulations describe unitary electronic dynamics and do not include explicit damping or relaxation mechanisms. Accordingly, the persistent excitonic polarization obtained after the pump should be understood within the simulated time window rather than as a quantitative prediction of the experimental exciton lifetime. Incorporating first-principles phonon-mediated dephasing^81^and finite exciton lifetimes would therefore enable a more realistic description of the persistence and decay of the excitonic polarization and the corresponding temporal evolution of the exciton-driven Floquet response.

## methods

### Exciton dynamics

In this section, we outline the methodology of our ab initio simulations. All calculations in this work are performed with the open-source, real-time, real-space OCTOPUS code^72^. To describe the exciton dynamics relevant to TrARPES, we employ real-time TDDFT in a real-space grid representation and solve the time-dependent Kohn-Sham (KS) equation in the velocity gauge. In atomic units, the time-dependent KS equation reads

1 $$i{\partial }_{t}{{{\Psi }}}_{n{{\bf{k}}}}({{\bf{r}}},t)=\left[\dfrac{1}{2}{\left(-i\nabla -\dfrac{{{\bf{A}}}(t)}{c}\right)}^{2}+{v}_{{{\rm{KS}}}}({{\bf{r}}},t)\right]{{{\Psi }}}_{n{{\bf{k}}}}({{\bf{r}}},t),$$

where Ψ_*n***k**_(**r**, *t*) denotes the KS orbital with band index *n* and crystal momentum **k,** *c* is the speed of light, and **A**(*t*) is the total vector potential. The time-dependent KS potential is given by

2 $${v}_{{{\rm{KS}}}}({{\bf{r}}},t)=\int{d}^{3}r^{\prime} \dfrac{\rho ({{\bf{r}}}^{\prime} ,t)}{| {{\bf{r}}}-{{\bf{r}}}^{\prime} | }+{v}_{{{\rm{xc}}}}[\rho ({{\bf{r}}},t)]+{v}_{{{\rm{ion}}}},$$

which consists of the Hartree potential, the exchange-correlation potential *v*_xc_ and the ionic potential *v*_ion_. The time-dependent electron density is given by *ρ*(**r**, *t*) = ∑_*n***k**_∣Ψ_*n***k**_(**r**, *t*)∣^2^. All technical details of the ground-state and time-dependent calculations are provided in the “First-principles calculation” subsection of the Methods.

To account for long-range electron-hole interactions and thereby describe bound excitons within real-time TDDFT, we employ the MLRC vector potential **A**_xc_(*t*). Its equation of motion is^67^

3 $${a}_{2}\dfrac{{d}^{2}}{d{t}^{2}}{{{\bf{A}}}}_{{{\rm{xc}}}}(t)+{a}_{0}{{{\bf{A}}}}_{{{\rm{xc}}}}(t)=\dfrac{4\pi c}{V}{{\bf{J}}}(t),$$

where **J**(*t*) is the macroscopic total current, *V* is the volume of the unit cell, and *a*_0_ and *a*_2_ are material-dependent parameters. The initial conditions are taken as **A**_xc_(0) = 0 and ${\dot{{{\bf{A}}}}}_{{{\rm{xc}}}}(0)=0$. Compared with the original LRC formulation^66^, this modified equation includes a finite-mass term proportional to **A**_xc_(*t*), which provides a numerically stable and efficient description of strongly bound excitons^67,68^. The total vector potential **A**(*t*) entering Eq. (1) is therefore decomposed as **A**(*t*) = **A**_ext_(*t*) + **A**_xc_(*t*), where **A**_ext_(*t*) denotes the external laser field.

For comparison, the TD-ALDA method we employed here follows the real-space, real-time formulation^73^. We represent the macroscopic polarization field with a spatially uniform, time-dependent vector potential **A**_ind_(*t*), whose dynamics satisfies

4 $$\dfrac{{d}^{2}}{d{t}^{2}}{{{\bf{A}}}}_{{{\rm{ind}}}}(t)=\dfrac{4\pi c}{V}{{\bf{J}}}(t).$$

The total vector potential entering the time-dependent Hamiltonian is then **A**(*t*) = **A**_ext_(*t*) + **A**_ind_(*t*). This self-consistent gauge-field formulation provides a real-time description of the macroscopic polarization response of the periodic solid. In the TD-ALDA calculation, no long-range exchange-correlation contribution is included, and therefore the field generated by the electronic response does not contain the excitonic contribution present in MLRC.

The macroscopic total current **J**(*t*) is obtained by integrating the gauge-invariant current density

5 $${{\bf{j}}}({{\bf{r}}},t)=\dfrac{1}{2i}\sum\limits_{n{{\bf{k}}}}{{{\Psi }}}_{n{{\bf{k}}}}^{* }({{\bf{r}}},t)\nabla {{{\Psi }}}_{n{{\bf{k}}}}({{\bf{r}}},t)+c.c.+\dfrac{1}{c}{{\bf{A}}}(t)\rho ({{\bf{r}}},t).$$

over the unit cell. From the resulting current, we evaluate the optical conductivity *σ*(*ω*) through the linear-response relation **J**(*ω*) = *σ*(*ω*)**E**(*ω*), where *ω* is the frequency, and **J**(*ω*) and **E**(*ω*) are the Fourier transforms of the macroscopic total current **J**(*t*) and electric field **E**(*t*), respectively. For the optical absorption calculation, we employ a *δ*-kick as our probe field, which means **E**(*t*) = **E**_0_*δ*(*t*) and **A**_ext_(*t*) = **A**_0_*θ*(*t*). As a result, we have **J**(*ω*) = *σ*(*ω*)**E**_0_, and the dielectric function is then obtained as *ϵ*(*ω*) = 1 + 4*πiσ*(*ω*)/*ω*, from which the optical absorption spectrum is calculated by taking the imaginary part of *ϵ*(*ω*).

### TrARPES simulations

To obtain the exciton-induced TrARPES intensity plots, we perform numerical pump-probe simulations in which the external laser field **A**_ext_(*t*) is decomposed as **A**_ext_(*t*) = **A**_pu_(*t*) + **A**_pr_(*t*). Here, **A**_pu_(*t*) and **A**_pr_(*t*) denote the pump and probe laser fields, respectively. For the resonant calculation, the pump frequency is chosen to match the exciton energy; an additional off-resonant case is considered for comparison. The probe pulse detects photoelectrons whose energy-momentum distribution is recorded in the TrARPES intensity plot. The pump field takes the form

6 $${{{\bf{A}}}}_{{{\rm{pu}}}}(t)={f}_{{{\rm{pu}}}}(t)\cos ({\omega }_{{{\rm{pu}}}}t){\hat{{{\bf{e}}}}}_{{{\rm{pu}}}}$$

where *ω*_pu_ is the pump frequency, ${\hat{{{\bf{e}}}}}_{{{\rm{pu}}}}$ is the pump polarization direction, and the envelope function *f*_pu_(*t*) is given by

7 $${f}_{{{\rm{pu}}}}(t)=\dfrac{c{E}_{{{\rm{pu}}}}}{{\omega }_{{{\rm{pu}}}}}{\sin }^{2}\left(\dfrac{\pi t}{{T}_{{{\rm{pu}}}}}\right){{\Theta }}({T}_{{{\rm{pu}}}}-t){{\Theta }}(t),$$

where *E*_pu_ is the magnitude of the electric field and *T*_pu_ is the pump-pulse duration. Here Θ(*t*) denotes the Heaviside step function, which ensures that the pump field **A**_pu_(*t*) in Eq. (6) is nonzero only within the interval *t* ∈ [0, *T*_pu_]. Similarly, the probe field is written as

8 $${{{\bf{A}}}}_{{{\rm{pr}}}}(t)={f}_{{{\rm{pr}}}}(t)\cos ({\omega }_{{{\rm{pr}}}}t){\hat{{{\bf{e}}}}}_{{{\rm{pr}}}}$$

with the probe envelope function

9 $${f}_{{{\rm{pr}}}}(t)=\dfrac{c{E}_{{{\rm{pr}}}}}{{\omega }_{{{\rm{pr}}}}}{\sin }^{2}\left[\dfrac{\pi (t-{T}_{{{\rm{pu}}}})}{{T}_{{{\rm{pr}}}}}\right]{{\Theta }}({T}_{{{\rm{pu}}}}+{T}_{{{\rm{pr}}}}-t){{\Theta }}(t-{T}_{{{\rm{pu}}}}),$$

where the probe parameters are defined analogously to those in Eq. (7). In the present setup, the pump and probe pulses do not overlap in time. This choice ensures that the features observed in the pump-probe spectra are not induced directly by the external pump field during photoemission, but instead arise from the oscillating excitonic field generated by the pump.

The TrARPES intensity plots are obtained directly from the propagated KS states using the t-SURFFP^70,71^. In this approach, the slab geometry is periodic in the in-plane directions and finite along the out-of-plane direction. The simulation region is partitioned into an inner region, where the electronic dynamics is described by the time-dependent KS Hamiltonian, and an asymptotic vacuum region, where the emitted electrons are approximated by Bloch–Volkov states. In the latter region, the Hamiltonian is written as

10 $${\hat{H}}_{{{\rm{V}}}}(t)=\dfrac{1}{2}{\left[-i\nabla -\dfrac{{{{\bf{A}}}}_{{{\rm{ext}}}}(t)}{c}\right]}^{2},$$

consistent with the velocity-gauge convention adopted throughout this work. The corresponding Bloch–Volkov states are expressed as

11 $${\chi }_{{{\bf{p}}}}({{\bf{r}}},t)={{\mathcal{N}}}{e}^{i{{\bf{p}}}\cdot {{\bf{r}}}}{e}^{-i{{\Phi }}({{\bf{p}}},t)},$$

with the Volkov phase

12 $${{\Phi }}({{\bf{p}}},t)=\dfrac{1}{2}\int_{0}^{t}d\tau {\left[{{\bf{p}}}-\dfrac{{{{\bf{A}}}}_{{{\rm{ext}}}}(\tau )}{c}\right]}^{2},$$

where **p** denotes the photoelectron momentum and ${{\mathcal{N}}}$ is a normalization factor.

The expansion coefficient of a time-propagated KS orbital Ψ_*n***k**_ onto a Bloch–Volkov state is obtained from the accumulated photoelectron flux through an analyzing surface *S* placed in the vacuum region,

13 $${b}_{n}({{\bf{p}}},t)=-\int_{0}^{t}d\tau {\oint }_{S}d{{\bf{s}}}\cdot \left\langle {\chi }_{{{\bf{p}}}}(\tau )\left\vert \hat{{{\bf{j}}}}(\tau )\right\vert {{{\Psi }}}_{n{{\bf{k}}}}(\tau )\right\rangle ,$$

where $\hat{{{\bf{j}}}}(t)$ is the velocity-gauge current-density operator. Finally, the momentum-resolved photoelectron probability is obtained from the squared modulus of these surface-flux coefficients,

14 $$P({{\bf{p}}})={{\lim }_{t\to \infty }}\dfrac{2}{N}{\sum }_{n = 1}^{N/2}{\left\vert {b}_{n}({{\bf{p}}},t)\right\vert }^{2},$$

where *N* denotes the number of escaped electrons. The t-SURFFP therefore provides the momentum-resolved photoemission signal directly from the time-dependent electron flux through the analyzing surface, without explicitly constructing continuum scattering states.

### First-principles calculation

Here we provide additional computational details for the simulations presented in the main text. We first summarize the ground-state calculation setup. The ground-state electronic structure is obtained by solving the KS equations self-consistently within DFT until the relative density convergence reaches 10^−9^. The local density approximation (LDA) and Hartwigsen-Goedecker-Hutter pseudopotentials^82^ are employed. In all calculations, the nonperiodic out-of-plane direction is described using a simulation box of 120 Bohr along the *z* axis. For monolayer hBN, the first Brillouin zone is sampled using a 12 × 12 × 1**k**-point grid. The simulation employs a real-space grid spacing of 0.34 Bohr and a lattice constant of *a* = 4.73Bohr. The resulting DFT band gap is 4.19 eV.

For the time-dependent calculations, we use the parameters *a*_2_ = − 13.96 and *a*_0_ = − 1 for monolayer hBN in Eq. (3). These parameters are found to produce reasonable exciton binding energies and a suitable description of ultrafast exciton dynamics. Throughout the time-dependent simulations, the scalar xc potential *v*_xc_ is treated within the adiabatic LDA, and the time step Δ*t* for the propagation scheme is 1.69 × 10^−3^ fs. For the TrARPES simulations, the analyzing surface used to evaluate the photoelectron flux is placed 30 Bohr away from the material along the nonperiodic direction. Complex absorbing boundaries are employed toward both edges of the simulation box to suppress unphysical reflections of the emitted electrons. The system is pumped by a linearly polarized laser pulse with ${\hat{{{\bf{e}}}}}_{{{\rm{pu}}}}=\hat{{{\bf{x}}}}$ and a peak intensity of *I*_pu_ = 4.996 × 10^10^W/cm^2^. The probe pulse is linearly polarized along ${\hat{{{\bf{e}}}}}_{{{\rm{pr}}}}=(\hat{{{\bf{x}}}}+\hat{{{\bf{z}}}})/\sqrt{2}$, with a peak intensity of *I*_pr_ = 3.982 × 10^9^W/cm^2^. The mixed in-plane/out-of-plane probe polarization is chosen to enhance the photoemission yield in the t-SURFFP calculation and follows the geometry previously benchmarked for monolayer hBN^71^. The Floquet band structures are obtained using our real-time Floquet-analysis code^20^. The one-period evolution operator is reconstructed directly from the time-propagated KS states as

15 $${U}_{ij}(t+2\pi /\omega ,t)=\langle {{{\Psi }}}_{i}(t)| {{{\Psi }}}_{j}(t+2\pi /\omega )\rangle ,$$

where *ℏω* = 3.6eV is the characteristic oscillation energy of the persistent excitonic polarization, consistent with the dominant excitonic resonance in the optical spectrum. Diagonalization of this operator yields the Floquet quasienergies and Floquet states. The Floquet states are subsequently decomposed into their harmonic components, which allows the quasienergy spectrum to be unfolded beyond the reduced Floquet zone and the corresponding Floquet weights to be evaluated. In the calculations shown in Fig. 4, harmonic Floquet components up to order *n* = ± 50 are included.

## Data Availability

The datasets generated and/or analyzed during the current study are not publicly available due to the large size of the raw real-time TDDFT datasets, but are available from the corresponding author on reasonable request.

## Code availability

The OCTOPUS code used to perform the simulations presented in the main text is available at https://gitlab.com/octopus-code/octopus.

## References

[1] de la Torre, A. et al. Colloquium: Nonthermal pathways to ultrafast control in quantum materials. *Rev. Mod. Phys.* **93**, 041002 (2021).

[2] Bao, C., Tang, P., Sun, D. & Zhou, S. Light-induced emergent phenomena in 2D materials and topological materials. *Nat. Rev. Phys.* **4**, 33–48 (2022).

[3] Xu, C. & Zong, A. Time-domain study of coupled collective excitations in quantum materials. *npj Quantum Mater.* **10**, 21 (2025).

[4] Bloch, J., Cavalleri, A., Galitski, V., Hafezi, M. & Rubio, A. Strongly correlated electron–photon systems. *Nature* **606**, 41–48 (2022). 10.1038/s41586-022-04726-w 35614214

[5] Oka, T. & Kitamura, S. Floquet engineering of quantum materials. *Annu. Rev. Condens. Matter Phys.* **10**, 387–408 (2019).

[6] Oka, T. & Aoki, H. Photovoltaic Hall effect in graphene. *Phys. Rev. B* **79**, 081406 (2009).

[7] Sentef, M. et al. Theory of Floquet band formation and local pseudospin textures in pump-probe photoemission of graphene. *Nat. Commun.* **6**, 7047 (2015). 10.1038/ncomms8047 25958840

[8] Chan, C.-K., Lee, P. A., Burch, K. S., Han, J. H. & Ran, Y. When chiral photons meet chiral fermions: photoinduced anomalous Hall effects in Weyl semimetals. *Phys. Rev. Lett.* **116**, 026805 (2016). 10.1103/PhysRevLett.116.026805 26824561

[9] Yan, Z. & Wang, Z. Tunable Weyl points in periodically driven nodal line semimetals. *Phys. Rev. Lett.* **117**, 087402 (2016). 10.1103/PhysRevLett.117.087402 27588882

[10] Claassen, M., Jia, C., Moritz, B. & Devereaux, T. P. All-optical materials design of chiral edge modes in transition-metal dichalcogenides. *Nat. Commun.* **7**, 13074 (2016). 10.1038/ncomms13074 PMC5062430 27721504

[11] De Giovannini, U., Hübener, H. & Rubio, A. Monitoring electron-photon dressing in WSe_2_. *Nano Lett.* **16**, 7993–7998 (2016). 10.1021/acs.nanolett.6b04419 27960498

[12] Hübener, H., Sentef, M. A., De Giovannini, U., Kemper, A. F. & Rubio, A. Creating stable Floquet-Weyl semimetals by laser-driving of 3D Dirac materials. *Nat. Commun.* **8**, 13940 (2017). 10.1038/ncomms13940 PMC5247574 28094286

[13] Liu, H., Sun, J.-T., Cheng, C., Liu, F. & Meng, S. Photoinduced nonequilibrium topological states in strained black phosphorus. *Phys. Rev. Lett.* **120**, 237403 (2018). 10.1103/PhysRevLett.120.237403 29932729

[14] Rudner, M. S. & Lindner, N. H. Band structure engineering and non-equilibrium dynamics in Floquet topological insulators. *Nat. Rev. Phys.* **2**, 229–244 (2020).

[15] Schüler, M. et al. How circular dichroism in time- and angle-resolved photoemission can be used to spectroscopically detect transient topological states in graphene. *Phys. Rev. X* **10**, 041013 (2020).

[16] Liu, X. et al. Floquet engineering of magnetism in topological insulator thin films. *Electron. Struct.* **5**, 024002 (2023).

[17] Neufeld, O., Hübener, H., Jotzu, G., De Giovannini, U. & Rubio, A. Band nonlinearity-enabled manipulation of Dirac nodes, Weyl cones, and valleytronics with intense linearly polarized light. *Nano Lett.* **23**, 7568–7575 (2023). 10.1021/acs.nanolett.3c02139 PMC10450813 37578460

[18] Fan, B., Duan, W., Rubio, A. & Tang, P. Chiral Floquet engineering on topological fermions in chiral crystals. *npj Quantum Mater.* **9**, 101 (2024).

[19] Gadge, K. et al. Comparative study of perturbative and nonequilibrium Green’s function approaches for Floquet sidebands in periodically driven quantum systems. *Phys. Rev. B* **114**, 125402 (2026).

[20] Li, R., Fan, B., De Giovannini, U., Hübener, H. & Rubio, A. First-principles Floquet analysis from real-time propagation. Preprint at 10.48550/arXiv.2607.04269 (2026).

[21] Sie, E. J. et al. Valley-selective optical Stark effect in monolayer WS_2_. *Nat. Mater.* **14**, 290–294 (2015). 10.1038/nmat4156 25502098

[22] Sie, E. J. et al. Large, valley-exclusive Bloch-Siegert shift in monolayer WS_2_. *Science* **355**, 1066–1069 (2017). 10.1126/science.aal2241 28280203

[23] Shan, J.-Y. et al. Giant modulation of optical nonlinearity by Floquet engineering. *Nature* **600**, 235–239 (2021). 10.1038/s41586-021-04051-8 34880426

[24] Zhang, X. et al. Light-induced electronic polarization in antiferromagnetic Cr_2_O_3_. *Nat. Mater.* **23**, 790–795 (2024). 10.1038/s41563-024-01852-w 38561519

[25] Tiwari, V., Korol, R. & Franco, I. Robust purely optical signatures of Floquet states in laser-dressed crystals. *Phys. Rev. Lett.* **135**, 186901 (2025). 10.1103/5ywx-7dbs 41247955

[26] Fan, B. et al. Floquet optical selection rules in black phosphorus. *Sci. Adv.* **11**, eadw2744 (2025). 10.1126/sciadv.adw2744 PMC12366682 40834080

[27] Lindner, N. H., Refael, G. & Galitski, V. Floquet topological insulator in semiconductor quantum wells. *Nat. Phys.* **7**, 490–495 (2011).

[28] McIver, J. W. et al. Light-induced anomalous Hall effect in graphene. *Nat. Phys.* **16**, 38–41 (2020). 10.1038/s41567-019-0698-y PMC6949120 31915458

[29] Park, S. et al. Steady Floquet-Andreev states probed by tunnelling spectroscopy. *Nature* **603**, 421–426 (2022). 10.1038/s41586-021-04364-8 35296842

[30] Zhu, T., Zhou, D., Wang, H., Wei, S.-H. & Ruan, J. Floquet odd-parity collinear magnets. *Phys. Rev. Lett.* **136**, 126704 (2026). 10.1103/7ywb-ml2q 41965045

[31] Huang, S. et al. Light-induced odd-parity magnetism in conventional antiferromagnetism. *Phys. Rev. Lett.* **136**, 126703 (2026). 10.1103/9346-9jpf 41965023

[32] Wang, Y., Steinberg, H., Jarillo-Herrero, P. & Gedik, N. Observation of Floquet-Bloch states on the surface of a topological insulator. *Science* **342**, 453–457 (2013). 10.1126/science.1239834 24159040

[33] Mahmood, F. et al. Selective scattering between Floquet–Bloch and Volkov states in a topological insulator. *Nat. Phys.* **12**, 306–310 (2016).

[34] Ito, S. et al. Build-up and dephasing of Floquet–Bloch bands on subcycle timescales. *Nature* **616**, 696–701 (2023). 10.1038/s41586-023-05850-x 37046087

[35] Bielinski, N. et al. Floquet–Bloch manipulation of the Dirac gap in a topological antiferromagnet. *Nat. Phys.* **21**, 458–463 (2025).

[36] Jabed, A. et al. Control of intervalley scattering in Bi_2_Te_3_ via temperature-dependent band renormalization. *npj Quantum Mater.* **11**, 12 (2026). 10.1038/s41535-025-00842-8 PMC12858402 41625017

[37] Choi, D. et al. Observation of Floquet–Bloch states in monolayer graphene. *Nat. Phys.* **21**, 1100–1105 (2025).

[38] Merboldt, M. et al. Observation of Floquet states in graphene. *Nat. Phys.* **21**, 1093–1099 (2025). 10.1038/s41567-025-02889-7 PMC12263427 40678614

[39] Wang, F. et al. Observation of Floquet-induced gap in graphene. *Nat. Mater.* **25**, 1140 (2026). 10.1038/s41563-026-02549-y 41872300

[40] Zhou, S. et al. Pseudospin-selective Floquet band engineering in black phosphorus. *Nature* **614**, 75–80 (2023). 10.1038/s41586-022-05610-3 36725995

[41] Zhou, S. et al. Floquet engineering of black phosphorus upon below-gap pumping. *Phys. Rev. Lett.* **131**, 116401 (2023). 10.1103/PhysRevLett.131.116401 37774306

[42] Bao, C. et al. Manipulating the symmetry of photon-dressed electronic states. *Nat. Commun.* **15**, 10535 (2024). 10.1038/s41467-024-54760-7 PMC11615328 39627195

[43] Bao, C. et al. Floquet-Volkov interference in a semiconductor. *Phys. Rev. B* **111**, L081106 (2025).

[44] Cai, X. et al. Occupation dynamics of Floquet–Volkov states and spectral sum rule. *Nano Lett.* **26**, 7034–7042 (2026). 10.1021/acs.nanolett.6c01340 42150054

[45] Aeschlimann, S. et al. Survival of Floquet-Bloch states in the presence of scattering. *Nano Lett.* **21**, 5028–5035 (2021). 10.1021/acs.nanolett.1c00801 PMC8227476 34082532

[46] Fragkos, S. et al. Floquet-Bloch valleytronics. *Nat. Commun.* **16**, 5799 (2025). 10.1038/s41467-025-61076-7 PMC12219864 40593831

[47] Duan, S. et al. Identification of metastable lattice distortion free charge density wave at photoinduced interface via TRARPES. *npj Quantum Mater.* **10**, 16 (2025).

[48] Fragkos, S. et al. Symmetry-driven Floquet engineering in multivalley SnS. Preprint at 10.48550/arXiv.2603.11878 (2026).

[49] Hübener, H., De Giovannini, U. & Rubio, A. Phonon driven Floquet matter. *Nano Lett.* **18**, 1535–1542 (2018). 10.1021/acs.nanolett.7b05391 29361223

[50] Chan, Y.-H., Qiu, D. Y., da Jornada, F. H. & Louie, S. G. Giant self-driven exciton-Floquet signatures in time-resolved photoemission spectroscopy of MoS_2_ from time-dependent GW approach. *Proc. Natl. Acad. Sci. USA* **120**, e2301957120 (2023). 10.1073/pnas.2301957120 PMC10410765 37523533

[51] Pareek, V. et al. Driving Floquet physics with excitonic fields. *Nat. Phys.* **22**, 209–217 (2026).

[52] Eskandari-asl, A. & Avella, A. Time-resolved ARPES in pumped excitonic systems: Floquet physics induced by excitonic fields. *Phys. Rev. B* **114**, 194304 (2026).

[53] Kobayashi, Y. et al. Floquet engineering of strongly driven excitons in monolayer tungsten disulfide. *Nat. Phys.* **19**, 171–176 (2023).

[54] Baykusheva, D. R. et al. Quantum control of Hubbard excitons. *Nat. Mater.* **25**, 937–943 (2026). 10.1038/s41563-026-02517-6 41803485

[55] Park, H., Park, N. & Lee, J. Novel quantum states of exciton–Floquet composites: Electron–hole entanglement and information. *Nano Lett.* **24**, 13192–13199 (2024). 10.1021/acs.nanolett.4c03100 39383357

[56] Xiao, T., Huang, T., Bao, C. & Sun, Z. Interaction effects on electronic Floquet spectra: Excitonic effects. *Phys. Rev. B* **112**, L161106 (2025).

[57] Park, H. & Lee, J. Floquet engineering of excitons in monolayer MoS_2_. *Phys. Rev. B* **111**, 184318 (2025).

[58] Onida, G., Reining, L. & Rubio, A. Electronic excitations: density-functional versus many-body Green’s-function approaches. *Rev. Mod. Phys.* **74**, 601 (2002).

[59] Rohlfing, M. & Louie, S. G. Electron-hole excitations in semiconductors and insulators. *Phys. Rev. Lett.* **81**, 2312 (1998).

[60] Rohlfing, M. & Louie, S. G. Electron-hole excitations and optical spectra from first principles. *Phys. Rev. B* **62**, 4927 (2000).

[61] Reining, L., Olevano, V., Rubio, A. & Onida, G. Excitonic effects in solids described by time-dependent density-functional theory. *Phys. Rev. Lett.* **88**, 066404 (2002). 10.1103/PhysRevLett.88.066404 11863831

[62] Marini, A., Del Sole, R. & Rubio, A. Bound excitons in time-dependent density-functional theory: Optical and energy-loss spectra. *Phys. Rev. Lett.* **91**, 256402 (2003). 10.1103/PhysRevLett.91.256402 14754131

[63] Botti, S. et al. Long-range contribution to the exchange-correlation kernel of time-dependent density functional theory. *Phys. Rev. B* **69**, 155112 (2004).

[64] Sharma, S., Dewhurst, J. K., Sanna, A. & Gross, E. K. U. Bootstrap approximation for the exchange-correlation kernel of time-dependent density-functional theory. *Phys. Rev. Lett.* **107**, 186401 (2011). 10.1103/PhysRevLett.107.186401 22107651

[65] Ullrich, C. Time-dependent density-functional theory: concepts and applications (Oxford University Press, 2012).

[66] Sun, J., Lee, C.-W., Kononov, A., Schleife, A. & Ullrich, C. A. Real-time exciton dynamics with time-dependent density-functional theory. *Phys. Rev. Lett.* **127**, 077401 (2021). 10.1103/PhysRevLett.127.077401 34459649

[67] Dewhurst, J., Gill, D., Shallcross, S. & Sharma, S. Kohn-Sham-Proca equations for ultrafast exciton dynamics. *Phys. Rev. B* **111**, L060302 (2025).

[68] Williams, J. R. & Ullrich, C. A. Stability of the long-range corrected exchange-correlation functional and the Proca procedural functional in time-dependent density-functional theory. *J. Chem. Theory Comput.* **21**, 4753–4768 (2025). 10.1021/acs.jctc.5c00122 40272478

[69] Chan, Y.-H., Qiu, D. Y., da Jornada, F. H. & Louie, S. G. Giant exciton-enhanced shift currents and direct current conduction with subbandgap photo excitations produced by many-electron interactions. *Proc. Natl. Acad. Sci. USA* **118**, e1906938118 (2021). 10.1073/pnas.1906938118 PMC8237677 34155136

[70] Tao, L. & Scrinzi, A. Photo-electron momentum spectra from minimal volumes: the time-dependent surface flux method. *New J. Phys.* **14**, 013021 (2012).

[71] De Giovannini, U., Hübener, H. & Rubio, A. A first-principles time-dependent density functional theory framework for spin and time-resolved angular-resolved photoelectron spectroscopy in periodic systems. *J. Chem. Theory Comput.* **13**, 265–273 (2017). 10.1021/acs.jctc.6b00897 27981832

[72] Tancogne-Dejean, N. et al. Octopus, a computational framework for exploring light-driven phenomena and quantum dynamics in extended and finite systems. *J. Chem. Phys.* **152**, 124119 (2020). 10.1063/1.5142502 32241132

[73] Bertsch, G. F., Iwata, J.-I., Rubio, A. & Yabana, K. Real-space, real-time method for the dielectric function. *Phys. Rev. B* **62**, 7998 (2000).

[74] Wirtz, L., Marini, A. & Rubio, A. Optical absorption of hexagonal Boron Nitride and BN nanotubes. *AIP Conf. Proc.* **786**, 391–395 (2005).

[75] Wang, J. et al. High-flux wavelength tunable XUV source in the 12–40.8 eV photon energy range with adjustable energy and time resolution for Tr-ARPES applications. *Opt. Express* **31**, 9854–9871 (2023). 10.1364/OE.484088 37157547

[76] Byun, Y.-M. & Ullrich, C. A. Assessment of long-range-corrected exchange-correlation kernels for solids: Accurate exciton binding energies via an empirically scaled bootstrap kernel. *Phys. Rev. B* **95**, 205136 (2017).

[77] Refaely-Abramson, S., Jain, M., Sharifzadeh, S., Neaton, J. B. & Kronik, L. Solid-state optical absorption from optimally tuned time-dependent range-separated hybrid density functional theory. *Phys. Rev. B* **92**, 081204(R) (2015).

[78] Sun, J., Yang, J. & Ullrich, C. A. Low-cost alternatives to the Bethe-Salpeter equation: Towards simple hybrid functionals for excitonic effects in solids. *Phys. Rev. Res.* **2**, 013091 (2020).

[79] Tran, V., Soklaski, R., Liang, Y. & Yang, L. Layer-controlled band gap and anisotropic excitons in few-layer black phosphorus. *Phys. Rev. B* **89**, 235319 (2014).

[80] Cao, T. et al., Valley-selective circular dichroism of monolayer molybdenum disulphide. *Nat. Commun.* **3**, 887 (2012). 10.1038/ncomms1882 PMC3621397 22673914

[81] Nie, Z. et al. First-principles electron-phonon scattering in real-time TDDFT. Preprint at 10.48550/arXiv.2607.17265 (2026).

[82] Hartwigsen, C., Goedecker, S. & Hutter, J. Relativistic separable dual-space Gaussian pseudopotentials from H to Rn. *Phys. Rev. B* **58**, 3641 (1998). 10.1103/physrevb.54.1703 9986014

